# The Role of Leisure-Time Physical Activity in the Change of Work-Related Stress (ERI) over Time

**DOI:** 10.3390/ijerph16234839

**Published:** 2019-12-02

**Authors:** Jean-Baptist du Prel, Johannes Siegrist, Daniela Borchart

**Affiliations:** 1 Department of Occupational Health Science, University of Wuppertal, 42119 Wuppertal, Germany; borchart@uni-wuppertal.de; 2 Institute of Medical Sociology, Heinrich-Heine-University Duesseldorf, 40225 Duesseldorf, Germany; Johannes.Siegrist@med.uni-duesseldorf.de

**Keywords:** leisure-time physical activity, work-related stress, effort-reward imbalance, older employees, longitudinal research

## Abstract

Background: Every second employee in Europe complains about work-related stress. Occupational stress due to an imbalance between efforts spent and rewards gained (effort-reward imbalance = ERI) is well investigated and it is associated with mental and physical health. A common guess is that leisure-time physical activity (LTPA) has beneficial effects on work-related stress. Yet, evidence in support of this assumption is weak, especially regarding ERI-stress. Longitudinal studies investigating the role of LTPA on ERI are missing. Therefore, this study aims to investigate the effect of LTPA on work-related stress by ERI over time. Methods: 3961 socially insured employees that were born in 1959 or 1965 and working in the first (t_1_: 2011) and second wave (t_2_: 2014) of the lidA-study were included. Work-related stress was measured by ERI, LTPA by the self-rated weekly frequency of physical activities. Besides the direct effect, a moderating effect of LTPA on ERI over time was tested in the multiple linear regression analysis. Results: The ERI at t_1_ was strongly associated with ERI at t_2_. While LTPA had no direct effect on ERI(t_2_), it was a significant moderator of ERI from t_1_ to t_2_: The higher the frequency of LTPA, the lower ERI was over time. This interaction of LTPA with ERI remained after adjustment for socio-demographic factors. Conclusions: The long-term moderating effect of LTPA on ERI is in agreement with former investigations on the role of LTPA on work-related stress, generally, and on its cross-sectional effect on ERI-stress, specifically. Some of Hill’s criteria of a causal association in epidemiology (biological gradient, temporality, consistency) support our findings. As LTPA has also been shown to exert a protective effect on health outcomes that are associated with ERI, the moderation of ERI by LTPA could partly explain this protective effect. Future observational and interventional studies are required to support our results over more than two age groups and study times.

## 1. Introduction

Work-related stress is widespread in modern societies. In Europe, every second employee reports to frequently suffer from stress at work, with every fourth being permanently adversely affected by it [1]. Work-related stress can be caused by different psychosocial or physical conditions. The focus here will be on work-related psychosocial stress. Chronic psychosocial stress at work has been measured with different models and it has been shown to be an important risk factor for health. The model of effort-reward imbalance (ERI) is one of the best-investigated psychosocial stress measures [2]. According to this model, occupational stress can be the result of a failed social reciprocity between efforts spent (e.g., work load, responsibility) and monetary as well as non-monetary rewards (esteem, job security, job promotion) received in turn [3]. ERI has been shown to be associated with common adverse health outcomes [4]. Recent evidence supports this notion for coronary heart disease [5], depressive disorders [6], and burnout [7].

Leisure-time physical activity (LTPA) is a further factor that is associated with common physical and mental diseases, and it has been shown to exert protective effects on coronary heart disease [8,9] and depression [10,11]. There is scientific evidence regarding the stress buffering effect of physical activity on health [12]. Yet, the effect of LTPA on work-related stress—one of the major stressors of today’s life—is not investigated as well as commonly assumed. There is some epidemiological evidence regarding the long-term beneficial effect of LTPA on work-related stress. One study reports a protective longitudinal effect of light and moderate-to-vigorous physical activity on self-reported stress level, when compared to a sedentary life style in health care and social insurance workers [10]. Furthermore, LTPA had a beneficial long-term effect on job strain in young adults (imbalance between high job demands and low job control, [13]) in their mid-years [14]. To our knowledge, the effect of LTPA on work-related stress measured by ERI over time was not investigated so far.

A negative effect of psychological stress on LTPA has been widely investigated, e.g., in a systematic review including 55 prospective studies [15], and it has also been observed for work-related stress [16]. Yet, the reverse association is less well studied [17].

With this study, we set out to analyze the effect of LTPA on work-related stress in terms of effort-reward imbalance in a longitudinal study design. 

## 2. Materials and Methods 

### 2.1. Participants and Procedure

We performed an exploratory investigation over two study waves in the frame of the German lidA(leben in der Arbeit)-study. lidA is a prospective cohort study on work, age, health, and work participation. Study subjects are employees who were born in 1959 or 1965 and are subject to social security contributions. The study sample is drawn by a two stage random sampling process from the ‘Integrated Employment Biographies’ (IEB) of the Federal Employment Agency in Germany. The IEB dataset includes all employees that are subject to social security in Germany. The primary response rate was 27.3% and the cooperation rate was 32.6% (RR5/COOP3), according to the American Association for Public Opinion Research standards [18]. A comprehensive description of the study design is given in the lidA-cohort profile [19]. A total of 3961 employees working in the first (t_1_ = 2011) and second wave (t_2_ = 2014) of the lidA-study were included in this analysis. 

### 2.2. Measures

#### 2.2.1. Work-Related Stress (ERI)

Work-related stress was measured based on the effort–reward imbalance model [3]. According to this model, an imbalance between efforts spent (e.g., responsibility or work load) and monetary and extra-monetary rewards (e.g., approval by superiors or colleagues) gained results in work-related stress. The complete ERI questionnaire contains 23 items in three sub-scales: ‘efforts’ (six items), ‘rewards’ (11 items), and ‘over-commitment’ (six items) [20]. The extrinsic component of effort–reward imbalance ratio (ERI-R) was calculated in this analysis by dividing the sub-scales ‘efforts’ and ‘rewards’ and adding a weighting factor of 6/11 to the denominator of the ERI-R to adjust for the different numbers of items in the nominator and the denominator to measure work-related stress. The value 1 defines a balance between efforts and rewards in this way, higher values different levels of work-related stress. Work-related stress was measured with the ERI-ratio at the first two study waves (2011, 2014) of the lidA-study. For description and bivariate analysis (Table 1), ERI-R at t_1_ and t_2_ were divided in classes of tertiles, in the multiple analysis we used the continuous ERI-R.

#### 2.2.2. Leisure-Time Physical Activity (LTPA)

Physical activity was assessed by a single question at the second study wave asking for the average weekly frequency of days with leisure-time physical activity (LTPA) for at least 30 min. leading to sweating or getting out of breath (no day with physical activity, less than one day, 1–2 days or three and more days per week).

#### 2.2.3. Covariates

The following socio-demographic variables were assumed to be potential confounders in the association between ERI-R and LTPA over time.

##### Age

The age of the employees was measured by the year of birth in two categories (1959, 1965) at t_1_.

##### Gender

Gender (male, female) was recorded by the interviewer at t_1_.

##### Education

Education, as a proxy of socioeconomic status, has the advantage to be a rather stable measure over time and include some aspects of job level as well because education is often a perquisite to attain a certain occupational position in Germany. Education was measured by a combined score of school education and vocational training, as recommended by the German Working Group Epidemiology [21]. School education and vocational training are both ranked from no degree at all to the highest (i.e., University entrance diploma for school education and University degree for vocational training), and the combined score of both measures ranges between one and eight points, accordingly. The educational score was then divided in three classes (low, middle, high). 

### 2.3. Statistical Analysis

Descriptions of employees characteristics bivariate analyses were performed with Cramer’s V. Multiple testing was performed with blockwise linear regression models. Moreover, we tested for effect modification of the association between ERI stress at t_1_ and t_2_ by LTPA at t_1_ in the multiple models. For this purpose, ERI-R at t_1_ and t_2_ were centered and the product term of ERI-R(t_1_)*LTPA was built. The percentage of missing data was particularly high for ERI-R over the two study waves (28%). The missing values were replaced by mean score imputation of the remaining items if there were no more than two items missing in the ERI-R scale. The data were analyzed with IBM Statistical Package for the Social Sciences (IBM SPSS Statistics for Windows, Version 25.0. Armonk, NY, USA: IBM Corp.).

### 2.4. Ethical Approval

The Ethics Committee of the University of Wuppertal approved the lidA-study on 5 December 2008.

## 3. Results

### 3.1. Description

ERI was relatively stable over the two study waves, as the group of those who were at the same stress level at the second time was relatively high at all stress levels (Table 1). 

### 3.2. Bivariate Analysis

Work-related stress measured with ERI-tertiles at study wave 1 (t_1_) was significantly associated with ERI-tertiles at study wave 2 (t_2_) (Table 1). This was the only significant association in the bivariate analysis. The association between gender and ERI at t_2_ was of borderline significance (*p* = 0.05) in the bivariate analysis, which was mainly due to the higher percentage in the upper ERI-tertile (high stress level) in women at t_2_.

### 3.3. Multivariate Analysis 

ERI-R at t_2_ was the dependent variable in the multivariate analysis (Table 2). First, we tested for the predictive value of ERI-R at t_1_ on ERI-R at t_2_ and then of LTPA while adjusting for each other and for covariates. In the multiple models, ERI-R at t_1_ was a significant predictor of ERI-R at t_2_. This association did not change after adjusting for physical activity and socio-demographic variables. LTPA itself had no significant predictive value on ERI-R at t_2_ after the adjustment for ERI-R at t_1_ and the covariates. 

**Figure 1:**
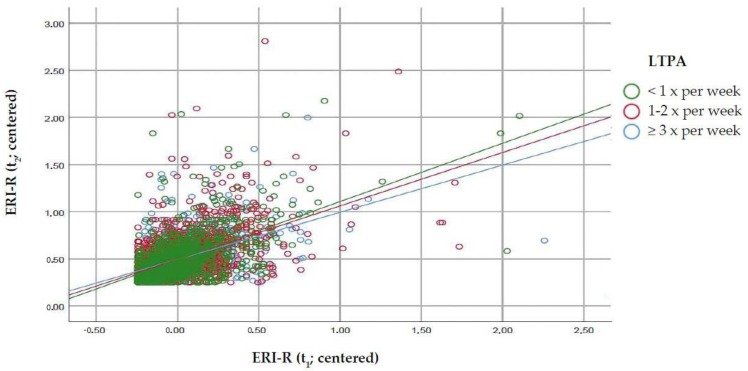
Effect modification of ERI-R over two study waves (t_1;_ t_2_) by LTPA (*n* = 3801) (Abbr.: ERI-R = effort-reward imbalance ratio; LTPA = leisure-time physical activity; t_1_ = study wave 1; t_2_ = study wave 2).

We then tested for interaction of the association of ERI-R over two times by leisure-time physical activity (Table 2, Figure 1). LTPA was a significant moderator of the association of ERI-R over two study waves (t_1_; t_2_). There was a dose-response relationship of the moderating effect of leisure-time physical activity on the association of ERI-R over time (t_1_ to t_2_). The higher the frequency of physical activity per week, the lower the ERI-R at the second study wave in comparison to the first wave. This interaction of LTPA with ERI-R was also observable after adjustment for LTPA itself and socio-demographic factors.

Gender was a significant predictor for work related stress (ERI-R) at t_2_ after adjustment for ERI-R at t_1_ with a higher risk for women.

## 4. Discussion

Work-related stress, as measured by the ERI-R at t_1_, was significantly associated with ERI-R at t_2_. LTPA was a significant moderator of the association of ERI-R over the two study waves (t_1_; t_2_): The higher the amount of weekly LTPA, the weaker the positive association between ERI-R at t_1_ and t_2_. The significant moderating effect of LTPA on ERI-R over time persisted after adjustment for socio-demographic variables. Of the socio-demographic covariates, only sex was a significant predictor of ERI-R over time to the disadvantage of women. 

The fact that we observed a dose-response relationship of the moderating effect of LTPA on work-related stress over time in the expected direction supports the assumption of a causal association, as discussed by Bradford Hill [22]. Moreover, these findings are in agreement with earlier studies addressing the beneficial effects of LTPA on work-related stress in general, and with a specific longitudinal study showing a moderating effect of LTPA on chronic work-related stress [23]. The consistency of independent study findings is a further criterion of epidemiologic causality, as discussed by Bradford Hill [22] (see also [24]). Education has the advantage of stability over time in comparison with other indicators of socio-economic status. This also holds true for the age cohorts born in 1959 and 1965 in our study, as there was a low probability of a fundamental change in educational status. In our longitudinal analysis, we could not find a significant association between the educational level and ERI-R.

Our results give support to a preventive effect of LTPA on work-related stress (ERI-R). According to Gerber and Pühse [12], a preventive effect on health is assumed if exercise contributes to a lower level of stress. This was shown, for instance, in the Copenhagen City Heart study, where increased exercise was followed by decreased perceived stress among participants [25]. Our finding of a dose-response relationship is in accordance with this result. 

While physiological measures of work-related stress, like heart rate variability (HRV), were not included in this study, its results are nevertheless in accordance with findings that pointed to an association of high physical activity with high HRV, a physiological correlate of low level of psychosocial stress [26]. Along this line of argument, fitness training might enhance the ability of cardiovascular systems to control responses to acute stressors as well as recover faster from stress [26,27]. 

Besides physiological adaptation to stress, it might also be that LTPA strengthens personal resources, such as self-esteem or social support [28], which contributes to a more favorable evaluation of one’s effort and reward at work. A study on the association between physical activity and work-related stress by ERI suggests that LTPA might strengthen the personal resilience to effort-reward imbalance [17]. While this study did not provide longitudinal evidence this could partly explain our findings of a beneficial effect of LTPA on work related stress over time. In the study of Gerber et al. [17], higher resilience to ERI was associated with less mental health problems. Our study did not test the so-called ‚stress-buffer hypothesis’ that assumes a buffering effect of physical activity on the health damaging effects of stress [28]. This effect has been also observed in the study of Klaperski et al. [23]. Further research along these lines is warranted although a direct test was not performed in our investigation. 

Last but not least, it can be meaningful to investigate the effect of LTPA on work-related stress over time, independently of a health-related outcome in the occupational context: ERI was also associated with other undesirable work-related effects, like higher intention to retire early [29], poor work performance, and reduction of work productivity [30]. Proving that LTPA had a positive effect on ERI over time means that it could also be protective against all of these adverse work-related outcomes associated with ERI. 

### Limitations

While the lidA-study has a large number of employees and is a prospective cohort study [19], there are some limitations of this analysis: the generalizability of our results to all employees might be limited, as only two age cohorts are included in the lidA-study and only those who are paying social security contributions (about 85% of all employees in these age groups). The relatively low response rate of 27.3% in the first study wave has to be seen from the vantage point of the observed high representativeness of our sample to all social secured employees in 16 different socio-demographic variables in comparison with the data of the ‘Integrated Employment Biographies’ (IEB), where the study sample has been drawn from [19,31,32]. The IEB dataset includes all employees subject to social security in Germany [19]. The loss to follow-up especially occurred in male employees, and missing values in effort-reward imbalance must be identified as clear limitations. 

Moreover, additional unmeasured factors may lead to a limited predictability of our results: We only included two study waves; therefore, time trends cannot be recognized in our analysis. Another limitation, the measurement of work-related stress, was restricted to the effort-reward imbalance model. Furthermore, LTPA was only measured with one item. With this measure, an examination of intricacies (e.g., type of activity) of LTPA or an analysis of whether LTPA is more effective in work-related stress reduction by ERI before or after work was not possible. Both aspects should be considered more detailed in future investigations. On balance, as a particular strength of this study, we applied a prospective cohort studies, based on a large sample, and we tested a relevant, not yet well studied, research hypothesis on the link between LTPA and ERI over time. 

## 5. Conclusions

LTPA might be helpful in reducing work-related stress in older employees suffering from an imbalance between high efforts spent and low rewards received in turn. As work-related stress is a widely prevalent phenomenon in modern working life and it exerts tangible negative effects on different health-related and work-related outcomes, our finding of an association between LTPA and ERI-R over time might be useful in planning sustainable interventions in occupational public health. LTPA might be beneficial in mitigating the health hazards of ERI (e.g., cardiovascular risk, depressive disorders) and their long-term consequences (e.g., loss of work force). LTPA should be promoted, therefore, in preventive activities aiming at stress reduction. Future prospective studies are required to further examine and extend our findings, applying objective data (e.g., measuring physical activity with accelerometer and work-related stress with biological markers, such as cortisol or heart rate variability). Moreover, intervention studies aimed at investigating the effect of LTPA on work-related stress should be implemented to strengthen the current base of evidence. 

## Figures and Tables

**Table 1:** Employees characteristics & bivariate results-(*N* = 3961).

Characteristics^(Time)^	Work-Related Stress (ERI-Tertiles)^(t^_2_^)^			*N* (*n*)	*p*-Value *
	High	Middle	Low		
**Work-related stress (ERI-tertiles)^(t^_1_^) a^**				3801	
high	62.00%	25.30%	12.70%	(1275)	
middle	29.60%	42.40%	28.00%	(1218)	**<0.0005**
low	10.60%	25.80%	63.60%	(1308)	
**Year of birth^(t^_1_^)^**				3905	
1959	35.00%	28.80%	36.20%	(1755)	0.069
1965	32.90%	32.20%	34.90%	(2150)	
**Sex^(t^_1_^)^**				3905	
female	35.50%	29.50%	35.00%	(2124)	0.05
male	31.90%	32.10%	36.00%	(1781)	
**Education^(t^_1_^) b^**				3880	
low	32.70%	28.50%	38.90%	(836)	
middle	34.20%	30.80%	35.00%	(2177)	0.163
high	33.90%	32.50%	33.60%	(867)	
**PA^(t^_2_^) c^**				3902	
<1 x per week	32.90%	29.70%	37.40%	(1121)	
1–2 x per week	33.60%	32.40%	34.00%	(1743)	0.192
≥3 x per week	35.10%	29.00%	35.90%	(1038)	

* Cramer’s V; significant results in bold print; ^a^ ERI = effort-reward imbalance (17 item version); ^b^ combined measure of school education and vocational training; ^c^ self-reported leisure-time physical activity ≥30 min with sweating or getting out of breath at t_2_.

**Table 2:** Blockwise linear regression of effort–reward imbalance ratio (ERI-R) at t_1_ and leisure-time physical activity (LTPA) onto ERI-R at t_2_ (*n* = 3771).

Variable^(Time)^	Model 1	Model 2	Model 3
	ß (95% CI)	ß (95% CI)	ß (95% CI)
**ERI-R^(t^_1_^)^**	0.57 (0.54; 0.60) ***	0.57 (0.54; 0.59] ***	0.56 [0.54; 0.59] ***
**LTPA^(t^_2_^)^**		0.002 [−0.01; 0.01]	0.003 [−0.005; 0.011]
ERI-R^(t^_1_^)^*LTPA^(t^_2_^)^		−0.06 [−0.1; −0.02] **	−0.06 [−0.1; −0.02] **
**Year of birth^(t^_1_^)^**			
1965 (Ref.)			
1959			−0.001 [−0.003; 0.001]
**Sex^(t^_1_^)^**			
male (Ref.)			
female			0.02 [0.01; 0.03] **
**Education^(t^_1_^)^**			−0.002 [−0.005; 0.002]

ERI-R = Effort-reward imbalance-ratio; LTPA = leisure-time physical activity; ^t^_1_ = study wave 1; ^t^_2_ = study wave 2; CI = confidence interval; Ref. = reference group; ERI-R^(t^_1_^)^*LTPA^(t^_2_^)^ = interaction term between ERI-R and LTPA; * *p* < 0.05; ** *p* < 0.01; *** *p* < 0.001.
